# Common patient experiences across three resource-oriented interventions for severe mental illness: a qualitative study in low-resource settings

**DOI:** 10.1186/s12888-022-04055-2

**Published:** 2022-06-18

**Authors:** Hana Sikira, Sabina Slatina Murga, Maja Muhić, Alma Džubur Kulenović, Stefan Priebe

**Affiliations:** 1 Department of Psychiatry, Clinical Centre University of Sarajevo, Sarajevo, Bosnia and Herzegovina; 2 Unit for Social and Community Psychiatry, WHO Collaborating Centre for Mental Health Services Development, Queen Mary University of London, London, UK

**Keywords:** Global mental health, Psychosocial interventions, Resource-oriented approach, LMICs, Solution-focused, Volunteer support, Family involvement, Severe mental illness

## Abstract

**Introduction:**

Resource-oriented interventions can be a low-cost option to improve care for patients with severe mental illnesses in low-resource settings. From 2018 to 2021 we conducted three randomized controlled trials testing resource-oriented interventions in Bosnia and Herzegovina (B&H), i.e. befriending through volunteers, multi-family groups, and improving patient-clinician meetings using the DIALOG+ intervention. All interventions were applied over 6 months and showed significant benefits for patients’ quality of life, social functioning, and symptom levels. In this study, we explore whether patient experiences point to common processes in these interventions.

**Methods:**

In-depth semi-structured interviews were conducted with 15 patients from each intervention, resulting in a total sample of 45 patients. Patients were purposively selected at the end of the interventions including patients with different levels of engagement and different outcomes. Interviews explored the experiences of patients and were audio-recorded, transcribed, and analysed using the thematic analysis framework proposed by Braun and Clark.

**Results:**

Three broad themes captured the overall experiences of patients receiving resource-oriented interventions: An increased confidence and agency in the treatment process; A new and unexpected experience in treatment; Concerns about the sustainability of the interventions.

**Conclusions:**

The findings suggest that the three interventions – although focusing on different relationships of the patients – lead to similar beneficial experiences. In addition to being novel in the context of the mental health care system in B&H, they empower patients to take a more active and confident role in treatment. Whilst strengthening patients’ agency in their treatment may be seen as a value in itself, it may also help to achieve significantly improved treatment outcomes. This shows promise for the implementation of these interventions in other low-resource countries with similar settings.

## Introduction

Psychosocial interventions that are low-cost and make use of a patient’s existing personal and social resources are an effective strategy to provide low-cost and effective health care for patients with severe mental illnesses in low-resource settings where there are often limited financial resources and trained professionals to provide specialized mental health services [1–4].

In Bosnia and Herzegovina, standard care for people living with psychiatric disorders consists mainly of pharmacotherapy, and treatment beyond medication, such as psychosocial interventions is usually not available.

Three such approaches, which utilize resources in communities, families, and routine clinical meetings that are often available in low-resource settings, include the DIALOG+ intervention, multi-family groups, and befriending programs. DIALOG+ is an intervention, incorporating the principles of solution-focused therapy and patient-centred communication designed to make routine patient-clinician meetings therapeutically effective. Supported by a tablet computer, it provides assessment, planning, intervention, and evaluation in one procedure [5]. Befriending programs pair unpaid volunteers from the community with a patient or group of patients for regular meetings with practical and social support [6]. Multi-family groups are based on the tradition of *trialogue* and psychosis seminars. They bring together several patients, members of their families or friends, and healthcare professionals in one group to promote mutual learning, support, and psychoeducation [7].

Previous research has shown significant benefits for patients with severe mental illness for all three- interventions in settings in high-income countries [5–9]. Three quantitative studies have been conducted in Bosnia and Herzegovina. All of these three interventions have been suggested to be effective. They share the general conceptual approach of mobilizing and utilizing existing resources of the patients, i.e. are resource rather than deficit oriented, but are very different in their form and practical aspects. The interventions have also shown to be both feasible and beneficial when implemented in low-resource settings. Both DIALOG+ [2] and befriending programmes [3] were found to have a positive impact on patients’ quality of life, and objective social situation and led to an improvement of symptoms. Family involvement was found to improve quality of life [10] and decrease the number of hospitalisations [11].

Previous qualitative research exploring the experiences of participating in resource-oriented interventions is limited and is restricted instead to the experiences of participating in single interventions, as opposed to exploring common processes across different resource-oriented interventions. Qualitative research to date indicates that participating in these interventions is a largely positive experience for patients. Specifically, in DIALOG+, patients reported that it promoted empowerment and allowed for greater self-reflection and expression [12]. Befriending through volunteers was shown to lead to benefits such as reducing stigma and building relationships [4, 13]. Family involvement interventions also were experienced positively by patients, providing a space conducive for shared learning to occur [7, 10].

This was an exploratory study about common experiences across three resource-oriented interventions that all are effective. The aim was to identify whether there were commonalities across the interventions for the people with severe mental illness, reflecting their shared conceptual approach, despite their very different formats and practical aspects of implementation.

The authors of this study are clinical-academic psychiatrists, psychiatry residents, mental health researchers from Bosnia and Herzegovina, and academic psychiatrists and psychologists from the UK of different ages. All were involved in the included studies which might have influenced their views towards rather positive perspectives.

## Methods

Current treatment options for outpatients in B&H are appointments with a psychiatrist once in 3–6 months. It usually consists of a short interview and medication prescription. This is the consequence of understaffed mental health facilities and persistent underinvestment in mental health care. Also, Bosnia and Herzegovina suffered 4 years of continuous conflict leading to an increased prevalence of trauma-related psychiatric disorders. At the same time, many psychiatric institutions, including long-term treatment facilities, were physically destroyed throughout the war. These two events led to a reform of mental health services since the conclusion of the conflict.

### Study design

This study explores the experiences of the patients who participated in three psychosocial interventions, conducted in Bosnia and Herzegovina (B&H). Three randomized controlled trials (RCTs) were conducted to test the efficacy of the DIALOG+, befriending through volunteers and multi-family groups for patients with severe mental illness. It is worthy of note, that the primary aim of the current study is to report on the experiences of a large sample of participants who took part in the same three psychosocial interventions, rather than a process evaluation of the original GLOBE studies per se. In-depth semi-structured interviews were conducted with 15 patients from each intervention, resulting in a total sample of 45 patients. This was qualitative study. The method we used is inductive thematic analysis.

The full protocol and findings from three original studies have been published elsewhere [1–3]. All three RCTs showed significant benefits for the intervention groups. Details of the trials and the findings have been published elsewhere 2,3,11.

### Recruitment and participants

Patients for the interviews were recruited from a larger sample of participants who were participating in a research program in Bosnia and Herzegovina, funded by the National Institute of Health Research in the United Kingdom (UK) (NIHR Global Health Research Group for Developing Psychosocial Interventions; GLOBE). The program included trials of DIALOG+, befriending through volunteers and multi-family groups in low-and-middle-income countries. All participants were recruited from the Clinical Centre, University of Sarajevo (KCUS).

Participants were eligible to participate in the interventions if they were over 18 years, could consent, had been receiving treatment at KCUS for a period of at least 6-months, and had a diagnosis of severe depression and anxiety (ICD:10 F30-F39, F40-F49) for the DIALOG+ study, or had received a diagnosis of psychosis (ICD10: F20-F29) for the multi-family groups and befriending trials.

Participants for the qualitative interviews were purposively selected from the intervention groups, to ensure demographic variability and to include both patients who were more or less engaged in the interventions. All studies received a positive ethical opinion from the Clinical Centre, University of Sarajevo School of Medicine Research Ethics Committee (Eticki Komitet) on 18/09/2018 and Queen Mary Ethics of Research Committee on 30/10/ 2018, ref.: QMERC2018/66. Written informed consent from participants was obtained for the GLOBE RCTs, which also included this interview study.

In total 45 participants, across the three interventions completed an interview at the end of the 6-month intervention period. All participants who were approached agreed to be interviewed.

In the befriending study 15 out of 33 patients who were in the intervention group were interviewed at the end of the intervention. The participants interviewed varied considerably in the number of befriending sessions they attended; ranging from 3–to 12 sessions, with a mean of 5 sessions attended. Interviews ranged in length from 30 to 90 minutes. The demographics of these 15 participants are presented in Table 1.

**Table 1:** Demographic characteristics of patients from DIALOG+, Volunteer support, and Family Involvement interventions

Intervention	DIALOG+	Volunteer support	Family Involvement
Mean age in years	44	38	42
Sex			
Male	2	8	12
Female	13	7	3
Marital status			
Single/Unmarried	5	10	4
Married	9	2	11
Divorced/separated	1	2	
Widow		1	
Education			
Primary or less	2	1	
Secondary	9	11	13
Tertiary or higher	4	3	2
Living with			
Alone	1	1	1
Family/friend	14	14	14
Employment			
Student	1	1	1
Full time	2	4	1
Part time		2	
Unemployed	11	7	12
Retired	1	1	1

In the multi-family group trial, 15 out of 36 patients from the intervention group were interviewed at the end of the 6-month intervention period. The mean number of sessions attended was 3.5 per patient (range 0–6). Patient attendance was 72% at the first session and 56% by the final session. The mean duration of each session was 95 minutes (range 60–110). Interviews ranged in length from 20 to 60 minutes. The demographics of the 15 participants are presented in Table 1.

In the DIALOG + trial, 15 out of 36 patients were interviewed at the end of the 6-month intervention period. The mean number of sessions attended was 3 (range 1–6). Interviews ranged in length from 20 to 90 minutes. The demographics of the 15 participants are presented in Table 1.

### Procedures

The topic guides for the semi-structured interviews were developed in collaboration with researchers from B&H, Colombia, Uganda, and the UK. The topic guides were designed to be open-ended, and guided by the participants, but included key topics to structure the conversation. Topics included: motivations for participating in the interventions, facilitators, barriers to the intervention, perceived effectiveness, implications, and suggestions to improve the program.

Participants who had finished the intervention were contacted by telephone and invited by a researcher to an interview. Interviews were conducted by three researchers in a variety of locations based on participant preference, including rooms at KCUS, or public locations if those locations were quiet and confidentiality could be ensured. Participants have been reimbursed 15 euros for being interviewed.

### Data analysis

Interviews were audio-recorded with two audio recorders and transcribed verbatim by the researchers who conducted the interviews. All transcripts were checked by other members of the research team for accuracy. Anonymized transcripts were analysed following the guidelines outlined by Braun and Clarke [14]. All three analysts were female, with a medical background, two of them in psychiatry. All researchers were trained in qualitative analysis.

All analysts read the full set of transcripts to familiarise themselves with the data, and five transcripts from each intervention were selected for line-by-line analysis, which was the most representative of the overall dataset. These five were selected by interviewers, based on participants’ demographic and engagement in the intervention. Line-by-line coding was completed independently by three analysts (DIALOG+: SS & HS; Befriending through volunteers: HS & MM; Multi-family groups: MM & HS).

A coding framework was developed based on a discussion of the initial codes and how they may be interlinked, specifically the two analysts wrote the initial codes on separate tables and explored how they could be grouped to form preliminary themes, based on emerging patterns. At this stage, any discrepancies, or disagreements were resolved.

Once the coding framework was developed, it was applied to the remaining transcripts. Any new codes not captured by the original framework were discussed with the larger research team, and the coding framework was modified to include these codes. This was inductive content analysis. By going through data, researchers developed themes and developed theories. Finally, the analysts reviewed and refined the themes.

## Results

Three themes were found to summarise the patient’s experience of participating across the three resource-oriented interventions: An increased confidence and agency in the treatment process; A new and unexpected experience in treatment; Concerns about the sustainability of the interventions. Various opinions were found during the analysis, but the most common and the most prominent ones were summarised in the themes above mentioned.

Overall, most of the patients have shown an affinity towards the interventions, which was followed by the improved symptoms of the mental health disorders and general affection towards new treatment option that was now more focused on the psychosocial aspects, rather than solely biological approach.

Many patients seemed to appreciate newly gotten space and time to share their problems and to have help in guidance or companionship in defining and learning how to handle these.

Quotations have also pointed out valuable information on facilitators and barriers in the conduction of the interventions which can be useful in the future implementation.

### An increased confidence and agency in the treatment process

Participants from all three studies reported that as a result of participating in the interventions they felt increased confidence and self-empowerment, which was followed by improvement in their symptoms, and the gaining of new habits and skills to deal with their condition.

*“Participating in the intervention allowed me to think more about myself, and I started to prioritize things, putting myself first, and I started to love and appreciate myself more, I learned to say no.” (DIALOG+).*

*“After taking part in the intervention it allowed me to talk to new people and even improved communication with people I already know. I started to hang out more with other people, that’s something I wasn’t used to. I became more confident socializing, even with people I’ve met only two or three times.” (Volunteer support study).*

*“I used to have huge problems with anxiety, always expecting a panic attack, everywhere I went, and I was nervous when around people. Now I am different. I go wherever I want, and talk to people. Now I’m able to attend appointments without my mom.” (DIALOG+).*

Participants also felt more empowered and confident to take an active role in their treatment. In the DIALOG + study, some participants described this was due to characteristics of the DIALOG + application. Some of the characteristics mentioned were DIALOG+‘s clear structure and solution-oriented approach**.** These characteristics seemed to be helpful for the patients in the sense of helping patients to more easily define the actual problems, break them down in part, and work on the problems step by step.

*“Each time I attended the appointment, I made some progress on the app, so it motivated me to accomplish all the tasks we made. Just having the goal written down makes it more likely to be accomplished, and in the end, that leads to the improvement.” (DIALOG+).*

*It is very useful to hear other peoples’ experiences, and hear about others’ lives and situations that happen to people, you have a chance to make a point. It’s always interesting how much I learn from these conversations when people share their stories. (Multi-family groups).*

The friendly atmosphere during the meetings seemed to be a huge step up from the usual setting. Patients described how this made them feel more equal with their clinician. The Family involvement and DIALOG+ interventions were also reported to improve patient-clinician communication, which in turn increased patients’ confidence in voicing their opinions, allowing them increased agency to participate more actively in their treatment.

*“I liked that the setting was relaxed, I felt like we are all equal, clinicians were not in their usual white coats, so that helped me to feel equal to them, and that allowed me to talk more openly and to ask the questions that I wanted to.” (Multi-family groups).*

*“The doctor and I, our communication became simpler, and our appointments became more comfortable during this study. It became easier for me to express my opinion in front of him. I wasn’t afraid I would say something wrong. This intervention taught me that there are no right or wrong answers. What is important is how I feel, and that I give my opinions to help myself.” (DIALOG+).*

### A new and unexpected experience in treatment

Several patients expressed that the three interventions provided a novel approach to the current treatment that they have been provided so far and that they have been used to. In low-resource settings, where resources are severely limited, many patients appreciated the addition of psychosocial interventions, as an adjunct to pharmacological treatment, which is currently the predominant treatment offer. Participants described several factors as a novelty in their treatment. For a start, just participating in the study was new to most, so inclusion was accompanied by scepticism.

As an obstacle, participants also mentioned meeting a completely new person with whom they should go through the intervention. In the befriending through volunteers, these were volunteers they saw for the first time, in the family intervention they would expose the family to the clinical environment for the first time and talk openly about their diagnoses, both to the family and unknown persons. In the terms of these, it was easier to enrol participants in the DIALOG study, taking into account that most of them already knew their clinician.

What participants have also generally described as a novelty factor in the treatment is better inclusion in treatment, more empowerment from their clinician, and better insight, which resulted in the increased agency in their treatment, which is in more detail explained in other themes.

Some of the patients have described greater empowerment from the clinician was reflected through the joined planning of the activities, helping in the analysis of the problems, not just in the treatment of the consequences.

*“From the time this project was introduced to me, I liked it for its novel approach, that offers a new way of dealing with our disorders, besides the pharmacotherapy. I felt like someone is paying attention to us, after all these years.” (Multi-family groups).*

‘*Yes, before the program I was worried about how it’s all going to look like, are we going to get along, what would we talk about... who that person would be. I was wondering why am I doing all this, but now I’m happy I did it. My expectations were much lower, and the experience was really good.. you know. I was thinking that our meeting would be some small talk, but it was surely better ... (Volunteer support).*

*‘To be honest, I didn’t expect anything, just the same visits to my psychologist as always. But it was different, longer, and better than I expected. (DIALOG+).*

During the qualitative interviews, many patients were prone to compare their expectations and later experience of the intervention. Most of them were genuinely surprised and satisfied with what they’ve got through this. Many patients were initially sceptical of the introduction of psychosocial interventions but later found the novelty of the approach to be beneficial in a way that pharmacological treatments alone are not. This often contained an element of surprise about an unexpected experience.

*“...in the beginning, I was very sceptical, but I changed my mind, and at this point, I can say [psychosocial interventions] are a great thing for people with mental illness. As a person who was hospitalized many times, it was hard to accept any type of treatment, but I can say this is a huge addition to meds. I think it is important to have something more than just medication... “(Volunteer support study).*

*“In the beginning I had reservations, I didn’t know what it was going to look like, but later, the atmosphere was good, it was not so clinical, no white coats (laugh), we even had some snacks during the meetings, it was much more relaxed [than usual clinical meetings].” (Multi-family groups).*

### Concerns about the sustainability of the interventions

Most of the patients felt that participating in the three resource-oriented interventions was a positive experience and that they would like to continue receiving the interventions in the future.

*“I would continue with these sessions. Even though I feel well now, it’s good to have a follow-up. It helped me to cope with everyday life, so that’s why I’d keep going to session if my clinician agreed.” (DIALOG+).*

*“It was helpful, for me at least […] I’m glad we were introduced to each other and matched this way. We’ve continued the contact and meetings after the intervention too.” (Volunteer support study).*

Motivations to continue with the family involvement intervention included the provision of a safe non-judgemental space, with other patients going through similar experiences, which made participants feel less isolated.

*“After participating in the intervention, both I and my husband felt we could handle things much better because we know that we’re not alone and this is happening not just to us.” (Multi-family groups).*

*“When you attend the session, you feel better, because you see you’re not all alone. My main motivation for attending the meetings was that I could speak about my feelings, and not feel like I have overwhelmed anyone, because we are all people with similar problems.” (Multi-family groups).*

Regarding the befriending through volunteers, many participants expressed that they were motivated to continue with the intervention due to the relationships that they had developed with their volunteers, including high levels of trust, reciprocity, and empathy.

*“It was okay for me to talk about my private life, I mean, she also opened to me, her life was tough too. I was expecting the relationship to be one-sided, but I’m glad she was honest with me too because that made our relationship a friendship - I’m glad she trusted me as much as I trusted her.” (Volunteer support study).*

Participants also discussed barriers that could affect engagement with the interventions. These varied from person to person based on individual factors but were often linked to practical factors, such as the need to optimize the location, frequency, and structure of meetings.

For example, in the DIALOG+ and Family involvement intervention, many participants were dissatisfied with the fact that the sessions were held in a clinical setting.

*“It felt stiff because the meetings were held in hospitals, this only reminds me of my hospitalization, I didn’t like the sessions being there.” (Multi-family groups).*

*“I think it would be better if these sessions were held in a less clinical space. I mean, it was okay for me, a friendlier space would add to a relaxed atmosphere.” (DIALOG+).*

The structure of the meetings was a concern for patients in the multi-family groups and the befriending program, with both expressing that a more flexible approach would benefit the interventions. In the multi-family groups, participants felt that having to decide on future discussion topics for meetings in advance made the meetings too rigid, and that time was wasted when an agreement about the topic for discussion, was unable to be reached. In the befriending trial, participants expressed they would have preferred to have a flexible approach, instead of a formal structure to the meetings, with the option to choose activities themselves.

*“I think it’s better for us if the moderator would come with the topic. It would be easier for everyone to agree, and we wouldn’t lose much time.” (Multi-family groups).*

*“It felt too rigid and stiff to have a structure for the meetings. It is important to have the flexibility to do whatever you want during the meetings. For me it was more important to have someone to push you to do the first step, just to get out of the house.” (Volunteer support study).*

## Discussion

This study explored the experiences of patients who participated in three psycho-social interventions in the context of low-resource settings. The commonality for all three interventions is that they use resources that are available to the patients, in terms of routine clinical meetings, friends and families, and volunteers available in communities. Although the interventions are distinct and work with different relationships of the patient, patients across the three interventions shared core experiences.

The findings suggest that all interventions have a substantial benefit in common. They all increase patients’ confidence and enable them to take a more active and empowered role in their treatment. This in turn led to further improvements as patients learned new skills to deal with their condition. For example, patients from the befriending trial reported that the increased confidence gained from participating in the intervention was a big step forward to resocialization and engagement with the community.

The increased confidence encouraged patients to participate in treatment decisions. This was in part attributed to the ability of the multi-family groups and DIALOG+ interventions to improve patient-clinician communication, and in part removed some of the hierarchy that was perceived as being prevalent in their usual routine clinical meetings. This provided a space where they felt more confident in voicing their opinions and participating more actively in their treatment. By increasing a patient’s agency to get actively involved in their health and treatment, these interventions could also potentially reduce the burden on clinicians to drive a patient’s treatment and recovery.

The participants interviewed in this study appreciated the novelty of the three different psychosocial interventions, which are vastly different from the current treatment approach, focusing largely on pharmacology. Although it is indicative that patients were positively surprised and receptive to the development of new approaches in their treatment, the novelty factor may fade over time if it became the norm.

Most patients expressed that they would like to continue receiving the interventions in the future, however they identified certain challenges which could affect the sustainability of such interventions. These factors should be considered to promote sustainability, especially if the novelty of the interventions was to wane, and engagement was to be affected. Most of the concerns about the sustainability of the interventions were about practical and organizational aspects. For example, patients felt that it was important that the intervention sessions should be held outside of hospital settings, as hospitals often conjured up negative associations and memories for them. Participants also held a preference for the intervention approach to be more flexible and less rigid. Considering such factors in the design and implementation of similar interventions in the future may promote greater levels of acceptance.

In all three interventions patients have built relationships, which might have been a facilitator in achieving a higher goal. In DIALOG+ patient-clinician relationship improvement was reported. In Family involvement, patients tended to bond with their family members easier after these meetings, and together to come to a better understanding. In befriending program, having a secure companion by their side induced some patients to socialize with other people. This might indicate a link between humanistic and therapeutically effect that might be achieved through the psychosocial interventions.

### Comparison with literature

Results from this study indicate that participating in resource-oriented psychosocial interventions can provide substantial benefits to the patient. Through empowering patients to become more actively involved in their treatment, and through learning new skills to deal with their conditions, treatment outcomes and symptom reduction may be achieved. This is by the quantitative results from the GLOBE studies, exploring the efficacy of these three psychosocial interventions within low-resource settings, which showed the interventions led to improved quality of life, symptom reduction, and better objective social functioning [2, 3, 11]. This study builds on the quantitative results by exploring what common processes may be underpinning the effects of the intervention.

About the DIALOG+ intervention, increased confidence and agency were in part attributable to the mechanisms inherent within the DIALOG+ intervention itself. A process evaluation of DIALOG+ conducted by Omer and colleagues [9] aimed to identify potential mechanisms in which DIALOG+ may be effective. Similar to this study, it was found that the comprehensive structure based on solution-focused therapy, provided a model which allows patients and clinicians to work together to address identified concerns. This promotes patients to become more aware of their situation and identify solutions, which in turn empowers them to become more active agents in their treatment [9].

The participants in this study discussed factors that might affect the sustainability of the intervention and offered recommendations to improve the interventions. Within the context of the DIALOG+ intervention and multi-family groups, a prevalent concern was related to the location of the intervention sessions, with a preference that they were conducted away from the hospital setting. This is in line with the original recommendations from the psychosis seminars model for multi-family groups, which recommends that meetings are held in a neutral space that does not hold therapeutic associations [15]. Participants also stated a preference for the interventions to be run with a greater level of flexibility. This mirrors the findings found from previous befriending studies, which indicate a preference for a more flexible approach, which does not rigidly focus on a set agenda of goal-setting, but instead provides a space where patients can feel listened to and supported [13, 16].

This study explored the experiences of patients in B&H. However, the findings are unlikely to be unique to this context. Qualitative findings exploring patients’ experiences of taking part in the same interventions in other low-resource settings suggest similar results. For example, in Pakistan, multi-family groups were similarly valued for providing a non-judgemental space that promoted mutual learning [10]. In Colombia, the befriending program led to confidence-building and personal growth [15].

### Strengths and limitations

To our knowledge, this is the first study exploring whether patient experiences point to common processes across three different resource-oriented interventions conducted within the context of low-resource settings. We expect these findings will enrich the understanding of the factors that may affect the uptake and acceptability of psychosocial interventions in other low resource settings, and be helpful in the design of future programs.

A strength of this study is the large sample size and that all participants who were invited agreed to complete the interview. Furthermore, we used a broad sampling strategy, purposively including patients with different levels of engagement with the interventions and demographic characteristics. This ensured that we got a varied range of opinions and experiences.

The study also has limitations. Firstly, all participants were recruited from the same clinic in Sarajevo, the largest city in B&H, and it is unknown whether experiences are representative of other settings, in particular rural areas with potentially different social conditions. Secondly, the analysis was completed by three researchers, who are psychiatry residents and had helped to set up and run the trials. They may therefore have been biased towards focusing more on the positive experiences of participating in the three interventions. To avoid bias, we ensured that researchers strictly follow the topic guides and that a broader group developed for each of the three studies. All of the audio recordings and transcripts were double-checked by two different persons.

### Implications

Psychosocial interventions explored in this study, have shown to be a valuable resource in the care of individuals living with mental illness in the context of Bosnia and Herzegovina. Understanding the experiences of patients has implications for the design and implementation of future programs to optimize psychosocial interventions in LMICs as a resource. Additional research is needed to specifically explore reasons for ‘drop-out’ from these programs and to identify factors that affect engagement. Also, this study implies that more research on psychosocial intervention is needed in LMIC, with the reflection on clinicians/caregivers/family members’ experiences. This study provides us with results, that can be used along with the results from the original GLOBE studies, in advocacy for the implementation of psychosocial intervention in routine mental health care in Bosnia and Herzegovina and LMICs.

## Conclusion

The results indicate that despite utilizing different relationships and resources that are available to the patient (i.e. through clinical, familial, and relationships through a volunteer), the three psychosocial interventions: DIALOG+, Family involvement, and Befriending through volunteers provided similar beneficial experiences. Most notably in that, they empowered patients to become more actively involved with their treatment. One may argue that such empowerment and increased agency are values in themselves and should be pursued in line with a humanistic and respectful form of mental health care. The findings of this study suggest not only that resource-oriented interventions in low-resource settings can achieve increased confidence and agency, but that these experiences are also central to improving treatment outcomes.

Potential barriers to intervention sustainability that were identified by participants appear surmountable and might be addressed by incorporating more flexibility into the design and implementation of similar interventions. The widely appreciated novelty factor of such interventions might fade over time, and it remains to be studied whether this would potentially affect engagement. However, in addition to the significantly improved outcomes achieved by the three interventions and their low costs, the overall positive experiences of increased patient confidence and agency identified in this study strengthen the case for a wider implementation for patients with severe mental illnesses in low resource settings.

## Data Availability

The datasets used and/or analysed during the current study will be available from the corresponding author on reasonable request.

## Abbreviations

B&H: Bosnia and Herzegovina
LMIC: Low-and middle-income countries
GLOBE: Global Mental Health Group on Developing Psycho-Social Interventions
KCUS: Clinical Centre University of Sarajevo
